# Editorial of the Special Issue “Targeted Therapies for Cancer”

**DOI:** 10.3390/biomedicines10051114

**Published:** 2022-05-11

**Authors:** Fabio Pastorino, Chiara Brignole

**Affiliations:** Laboratorio di Terapie Sperimentali in Oncologia, IRCCS Istituto Giannina Gaslini, 16147 Genoa, Italy

Cancer, the second leading cause of death worldwide, continues to represent an impressive challenge for researchers and clinicians. Indeed, radiotherapy and chemotherapy are still the main therapeutic options, associated, however, with undesirable toxicity. Furthermore, chemotherapy is frequently accompanied by the appearance of the drug resistance, which, in turn, further limits the overall success of the therapy. In the last decades, much work has been devoted to the development and/or implementation of strategies of cellular and molecular targeted therapies. For instance, Tamoxifen, which specifically targets the estrogen receptor in breast cancer, is the first drug approved by the Food and Drug administration (FDA) in 1977 as cellular targeted therapy for the treatment of metastatic breast-cancer-affected patients [1] and continues to be one of the most widely used treatment options. Dinutuximab, a monoclonal antibody (mAb) targeting the disialoganglioside GD2, expressed by neuroectodermal-derived tumors, was approved by the FDA in 2015 for the treatment of high-risk neuroblastoma-affected patients [2]. On the other hand, most efforts have been so far spent to identify, and target, specific molecular targets involved in cancer initiation and progression and, consequently, suitable for therapeutic purposes. Several small molecule inhibitors have been developed and tested in preclinical studies and, to date, 89 of them have been approved by the FDA [3]. Among these, imatinib, a Bcr/Abl1 inhibitor, was the first inhibitor to be approved in 2001 [3]; crizotinib is a first-generation ALK inhibitor approved in 2011 [3]; sirolimus is a rapamycin analog, which targets the PI3K/mTOR pathway, approved in 2015 [3]. Noteworthy, whole-exome sequencing (WES) technology has recently quickened the identification of potentially actionable gene mutations, leading to a step forward in so-called, personalized, patient-tailored therapy [4]. Furthermore, much progress have been made in the field of immunotherapy with several mAbs, Antibody-Drug Conjugates (ADCs) and immunotoxins being investigated in preclinical studies, some in ongoing clinical trials, and few of them approved by the FDA for their clinical use [5,6]. Finally, the identification of novel cellular targets drove the development of targeted delivery systems (e.g., liposomes, dendrimers, micelles), which exploit tumor-associated antigens and tumor-specific antigens to selectively deliver their payload to the diseased tissues [7].

In the “Targeted Therapies for Cancer” Special Issue, six original articles, one perspective article and three reviews intend to provide an overview on the state-of-the-art of targeted therapies for cancer, covering some of the aspects of this broad field. This Editorial briefly summarizes findings and highlights derived from the published manuscripts.

Jebali and colleagues [8] focused their study on RICTOR, a rapamycin-insensitive companion of mTOR, and its involvement in melanoma tumorigenesis and resistance to BRAF inhibitors. The authors demonstrated that RICTOR is overexpressed in metastatic melanoma, compared to primary melanoma, and it is associated with a worse overall survival in The Cancer Genome Atlas (TCGA) dataset cohort of melanoma patients. RICTOR overexpression also correlates with the overexpression of BRAF. In BRAF-mutated melanoma cell lines, the induced overexpression of RICTOR promotes the proliferation of spheroids culture by stimulating cells endowed with stemness capacity. In addition, RICTOR is implicated in the development of melanoma resistance to BRAF inhibitors. With this study, authors indicated RICTOR as a potential therapeutic target in melanoma, due to its pivotal role in BRAF-dependent melanoma development and resistance to therapy.

The involvement of Lamin A/C (LMNA gene) in the tumorigenesis and progression of glioblastoma multiforme (GBM) was instead investigated by Gatti and colleagues [9]. It was demonstrated that the expression levels of Lamin A/C correlate with a reduced overall survival in patients affected by almost all subtypes of GBM in the cohort of the TCGA dataset. The expression of the LMNA gene positively correlates with genes belonging to the regulation of cancer-related pathways, in particular cell adhesion and migration. In vitro, the forced overexpression of the LMNA gene in GBM cell lines was associated with a more aggressive phenotype, underlined by increased capability of colony formation, migration as well as increased tumor take and growth when injected in mice. Overall, the manuscript indicates Laminin A/C as a good biomarker for GBM-affected patient’s stratification and also proposes Laminin A/C as a valuable therapeutic target. Noteworthy, also in this study, RICTOR plays an important role, here mediating LMNA-induced aggressiveness and tumorigenicity in the GBM model.

In addition, the study conducted by Yu and colleagues [10] is focused on GBM, whose prognosis is still very poor. The activity of NSC13902, a small molecule compound targeting the atypical protein kinase RIOK2, overexpressed in GBM, was here investigated. The authors demonstrated that NSC139021 efficiently inhibited GBM cell proliferation through a RIOK2-independent manner. It was also shown that the Skp2-p27/p21-Cyclin E/CDK2-pRb signaling pathway was involved. In vivo, NSC139021 administration delayed the tumor growth of human and mouse GBM, indicating NSC139021 as a potential chemotherapeutic for GBM and for other tumors, such as gastric cancer and neuroblastoma (NB), where the same signaling pathway is involved. 

The implication of Nuclear factor erythroid-2 related factor-2 (Nrf2) in breast cancer (BC) responses to chemotherapy was instead investigated by Bovilla and colleagues [11]. The expression and the activity of Nrf2 were evaluated and found elevated in BC cell lines, in advanced grade II and III BC as well as in triple-negative BC tumors. The selective silencing of Nrf2, by the use of siRNAs, reduced BC cells’ viability. Furthermore, Nrf2 knockdown also sensitized BC cells to treatment with cisplatin. The pharmacological inhibition of Nrf2, by means of Brusatol, a natural compound isolated from Brucea javanica, determined a reduction in cell viability, cell cycle arrest and inhibition of cell migration. Furthermore, Brusatol reduced tumor growth in vivo and increased lymphocytes trafficking toward the tumor mass. The above findings highlight Nrf2 as a potential therapeutic target in BC.

Eliseev and colleagues [12] focused their studies on the ErbB3 receptor, which plays a pivotal role conferring resistance to the pharmacological inhibition of EGFR and HER2 receptor tyrosine kinases in cancer. A new class of heavy-chain Abs, differing from the classical ones because they lack the light chains and endowed with a variable chain represented by a single-immunoglobulin domain, was here investigated. These Abs were discovered in Llama, immunized with the extracellular domain of ErbB3. Three different single-domain Abs were purified and characterized and the two endowed with the higher affinity for ErbB3 were investigated for their effectiveness against ErbB3-positive cancer cell lines. Both Abs inhibited the ErbB3-driven proliferation of two breast cancer cell lines, also overexpressing ErbB2. These Abs, which recognize different epitopes of ErbB3 and have a non-competitive binding to the receptor, work via different mechanisms, suggesting their potential synergism if given in combination. The authors also propose that these two novel Abs could be suitable for the design of bi-specific Abs against ErbB2/ErbB3-positive cancers and might also represent a template for the production of antibody-drug conjugates.

Rango and colleagues [13] investigated the anticancer effectiveness of the pyrazolo[3,4-d]pyrimidine derivative si306 when encapsulated within NB-recognizing liposomes. Si306, a competitive inhibitor of c-Src tyrosine kinase, is characterized by sub-optimal aqueous solubility, thus limiting its therapeutic applicability. To overcome this problem, the authors encapsulated si306 within pegylated stealth liposomes and decorated them with anti-GD2 mAbs, specifically targeting disialoganglioside GD2-expressing NB cells. This formulation led to strong antitumor activity, in terms of the reduction in NB cells’ viability in vitro. These results were confirmed in a clinically relevant mouse model of NB, where the administration of GD2-targeted liposomes-encapsulating si306 significantly enhanced the life span of tumor-bearing mice, compared to control mice and mice treated with the untargeted formulation. This work highlights the potential benefit derived from the application of targeted nanodevices in cancer.

Fresnais and colleagues [14] presented a perspective article intended to the good understanding of the mode of action and monitoring of temozolomide (TZM), which represents a standard of care for GBM treatment. In particular, the authors summarized the known ADME (adsorption, distribution, metabolism, excretion) parameters and the sites of action (SOAs) of TZM. Specifically, mass-spectrometry-related methodologies are described and discussed in the text. The importance of TZM monitoring is highlighted by the authors: (1) for drug development and testing, to better control events related to the administration of TZM in combination with a new drug during a clinical trial; (2) for precision medicine approaches, with the aim to control if TZM is able to reach SOAs in sufficient amounts and to exert its expected affects. Overall, this manuscript defines a good monitoring workflow, which could be beneficial for both drug development and precision medicine approach applications.

A new and interesting approach of cancer therapy consisting in the targeted osmotic lysis (TOL) was presented by the review article of Gould and Paul [15]. This approach is proposed for the treatment of advanced-stage carcinomas. Epithelially derived cancers overexpress voltage-gated sodium channels (VGSCs) and Na+/K+-ATPase, which confer tumor cells the ability to invade normal tissues and to metastasize. Furthermore, VGSC expression is directly related to the grade of malignancy. Instead of blocking the expression or hindering the function of VGSCs, the TOL technology relies on the enhancement of VGSCs functionality and the simultaneous blockade of sodium pumping mechanism. In this way, the increased amount of sodium ions inside the cells leads water to passively enter the cells following sodium by osmosis. Under these conditions, tumor cells undergo lysis, while normal cells are spared from damage because they express VGSCs at significant lower levels compared to carcinomas. The authors presented some examples of validating TOL technology effectiveness, both in in vitro and in vivo mouse models of BC. Moreover, TOL treatment was also administered to dogs and cats for the treatment of a variety of advanced-stage carcinomas, revealing its efficacy. Noteworthy, a patient suffering from late-stage squamous cell carcinoma of the cervix was treated with TOL technology under an Emergency Use protocol, and the post-treatment survival exceeded expectation. This approach warrants further investigations but seems to represent an alternative strategy, safe and well tolerated, to treat advanced-stage carcinomas.

Advances and limitations in the development of Antibody-Drug Conjugates (ADCs) are discussed in the review article from Mckertish and Kayser [16]. ADCs are composed of a toxic drug, linked to a specific Ab directed against the target cells. At present, more than 100 ADCs are in ongoing clinical trials, and for a few of them, the phase III trial gave promising results. Noteworthy, nine ADCs have been also approved for clinical use. The authors point out what can be taken into consideration for the successful development of an ADC. Linker’s type (cleavable vs non-cleavable, both of them endowed with pro and cons) and payloads are the most important issues to be considered. The authors also outline the main limitations and challenges associated with the development of ADCs and to their clinical application: (1) off-target toxicities; (2) clearance rates; (3) protein aggregation; and (4) drug resistance development. It looks clear that ADCs represent an appealing targeted treatment approach; however, further studies are needed to overcome the main limitations associated with their broad and successful clinical application.

The review article by Wolf [17] focuses, instead, on the development of targeted toxins for the treatment of prostate cancer (PC), which represents the fifth leading cause of cancer deaths in men worldwide. The chemical structure of the targeted toxins relies on a binding domain (e.g., antibody, cytokine, hormone, growth factor) chemically linked to a toxic domain (toxins, mostly derived from bacteria and plants). At present, the FDA has approved only three targeted toxins and only for the treatment of hematological malignancies; others are in ongoing clinical testing for solid tumors. Although several targeted toxins have been developed to treat PC, with EGFR and PSMA being the most frequently targeted antigens, none of them have been tested in clinical trials against PC. This is due to several limitations (e.g., immunogenicity, low antigen binding, endosomal entrapment of the targeted toxins) that need to be solved. An overview of the possibilities to overcome and address these challenges is given: (1) to reduce the immunogenicity of the binding and toxic domains; (2) to improve the intracellular trafficking of the targeted toxins by avoiding degradation; and (3) to reduce off-target effects and enhance tumor penetration and affinity. It seems that the way to reach the clinical approval has been opened, and future investigations will hopefully lead to the final goal.
